# Temperature-dependent folding allows stable dimerization of secretory and virus-associated E proteins of Dengue and Zika viruses in mammalian cells

**DOI:** 10.1038/s41598-017-01097-5

**Published:** 2017-04-19

**Authors:** J. L. Slon Campos, S. Marchese, J. Rana, M. Mossenta, M. Poggianella, M. Bestagno, O. R. Burrone

**Affiliations:** Molecular Immunology Group, International Centre for Genetic Engineering and Biotechnology, 34149 Trieste, Italy

## Abstract

Dengue and Zika are two of the most important human viral pathogens worldwide. In both cases, the envelope glycoprotein E is the main target of the antibody response. Recently, new complex quaternary epitopes were identified which are the consequence of the arrangement of the antiparallel E dimers on the viral surface. Such epitopes can be exploited to develop more efficient cross-neutralizing vaccines. Here we describe a successful covalent stabilization of E dimers from Dengue and Zika viruses in mammalian cells. Folding and dimerization of secretory E was found to be strongly dependent on temperature but independent of PrM co-expression. In addition, we found that, due to the close relationship between flaviviruses, Dengue and Zika viruses E proteins can form heterodimers and assemble into mosaic viral particles. Finally, we present new virus-free analytical platforms to study and screen antibody responses against Dengue and Zika, which allow for differentiation of epitopes restricted to specific domains, dimers and higher order arrangements of E.

## Introduction

The Flaviviridae family includes some of the most important arthropod-borne human pathogens such as Dengue virus (DENV), Zika virus (ZIKV), West Nile virus (WNV) and Yellow fever virus (YFV)^1^. Over the last three decades, DENV infections have increased at an unprecedented rate and are now one of the most important human infectious diseases worldwide, with an estimated annual incidence of 390 million cases, 100 million of which show clinical manifestations of the infection^2^. Until recently, ZIKV infections were sporadic, mostly asymptomatic and restricted to specific regions in Africa and Southeast Asia^3^. However, since the 2007 outbreak in the South Pacific islands, the virus has spread rapidly to a global scale that almost matches the distribution of dengue and is now associated with serious neurological and developmental pathologies^3, 4^.

Like all flaviviruses, DENV and ZIKV are enveloped viruses with a ≈11 Kb single-stranded, positive-sense RNA genome which codes for a single viral polyprotein that is processed into 10 mature viral proteins: 3 structural (Capsid (C), pre-membrane (PrM) and envelope glycoprotein (E)) and 7 non-structural (NS) proteins (NS1, -2A, -2B, -3, -4A, -4B and -5)^5^. Of these, the E glycoprotein, a class II viral membrane fusion protein, covers almost the entire surface of the viral particle, serving pivotal functions during viral assembly and internalization^6^. Based on the sequence of this antigenic protein, DENV is composed of 4 closely related serotypes (DENV1, DENV2, DENV3 and DENV4) while ZIKV has been shown to involve a single serotype^7^.

On the surface of the mature viral particle, E folds into an elongated rod-like structure forming 90 antiparallel homodimers, organized in 30 rafts, each composed of 3 parallel E dimers distributed in a herringbone-like configuration^8^. The E protein ectodomain, also termed soluble E (sE), is formed by three different structural domains named DI, DII and DIII. DI has an 8-stranded β-barrel structure and is located at the center of the monomer with an axis parallel to the viral membrane^9^. DII is formed by two coding segments that fold together in an elongated finger-like structure with a highly stable core composed of an antiparallel 5-stranded β-sheet and 2 α-helices from which an elongated 3-stranded β-sheet expands distally, forming two loops^10^. The most distal one (cd loop) carries the hydrophobic glycine-rich fusion loop (FL) that is highly conserved in related flaviviruses^11^. In addition, DII provides the surface where some of the main interactions that drive E antiparallel dimerization occur^12^. DIII is an Ig-like β-barrel domain that shows variability among the different serotypes and has been implicated in the initial interaction to cellular receptors^13^. In addition, the 4 loosely packed peptide strands that connect DI and DII form a functional domain named Hinge region, which provides the flexibility needed for E trimerization^14^. In this pH-induced conformational reorganization of the viral surface, DII drives E reorientation from a horizontal antiparallel dimer into a vertical parallel trimer exposing the FL outwards to initiate the fusion process^15^.

It has been proposed that during viral assembly the PrM protein interacts with the nascent E protein to assist its proper folding and remains bound to the fusion loop during its transit through the secretory pathway, thus preventing premature fusion of the viral particle with the ER membrane^16^. As the nascent virion enters the Golgi network, Pr is cleaved by the host-encoded furin protease and remains attached to E until the virion is secreted^17^. Upon release from the cell, the neutral pH of the extracellular environment stabilizes E dimers and promotes dissociation of Pr^18^.

As described for both DENV and ZIKV, E protein is also the main target of the human antibody response upon infection^19, 20^. Neutralizing epitopes have been described on all three domains but the immune response is heavily dominated by poorly neutralizing and highly cross-reactive antibodies against epitopes on DI and DII (DI/DII)^21^. On the other hand, antibodies against DIII are generally described to be highly neutralizing and usually serotype specific^22, 23^. However, the contribution of these antibodies to the overall immune response in infected individuals appears to be very limited^24^.

The symmetric organization of E proteins on the virus creates new quaternary epitopes involving two or more E molecules found only on the surface of the intact virion. Recent data indicates that these epitopes are targeted by the majority of strong neutralizing antibodies found in the sera of infected patients^25^. Recently, a new type of potently neutralizing antibodies, recognizing epitopes restricted to the interface between two E proteins in the head-to-tail dimer conformation, has been described^26^. These dimer-dependent epitopes (named E-dimer epitope, EDE) do not require higher-order structural arrangements beyond the dimeric conformation of E, and appear to be highly conserved among flaviviruses^27, 28^. Antibodies against these epitopes were shown to have strong cross-neutralizing activity not only among DENV serotypes, but also against ZIKV^29, 30^.

The development of an efficient vaccine against DENV and ZIKV has become a high priority. One of the main obstacles for developing viable DENV vaccines is the risk of enhancing infection through the mechanism known as antibody-dependent enhancement of infection (ADE). In ADE, opsonization of viral particles by antibodies in non-neutralizing conditions leads to an increased Fcγ receptor-mediated uptake by monocytes and macrophages, which results in an increased and more severe infection^31^. Recently, cross-reactive antibodies against conserved epitopes in DI and DII, particularly those against the FL, were shown to enhance infection of other related flaviviruses like ZIKV and YFV^32–34^. The discovery of complex quaternary epitopes on DENV E protein dimeric structure, some of them conserved for instance in ZIKV, and recognized by antibodies with strong neutralization activity, has opened the possibility of developing a safe universal vaccine. However, the isolation of the precise epitopes to induce strong cross-neutralizing antibodies while avoiding induction of unwanted anti-DI/DII antibodies is a complex and yet unresolved task^35^.

With the aim of understanding and characterizing the dynamics involved in E dimerization, we describe a successful covalent stabilization of E dimers of DENV and ZIKV, along with a detailed biochemical analysis of the expression, secretion and folding in mammalian cells that provide a platform to facilitate the development of E dimer-based genetic or subunit vaccines. Moreover, we demonstrate that E dimerization within the ER lumen is independent of PrM but highly dependent on temperature, and show that because of their close structural and functional characteristics, DENV and ZIKV E proteins can interact to form heterodimers and assemble into mosaic viral particles.

## Results

### Temperature-dependent secretion of a covalently stabilized dimeric sE

**Figure 1:**
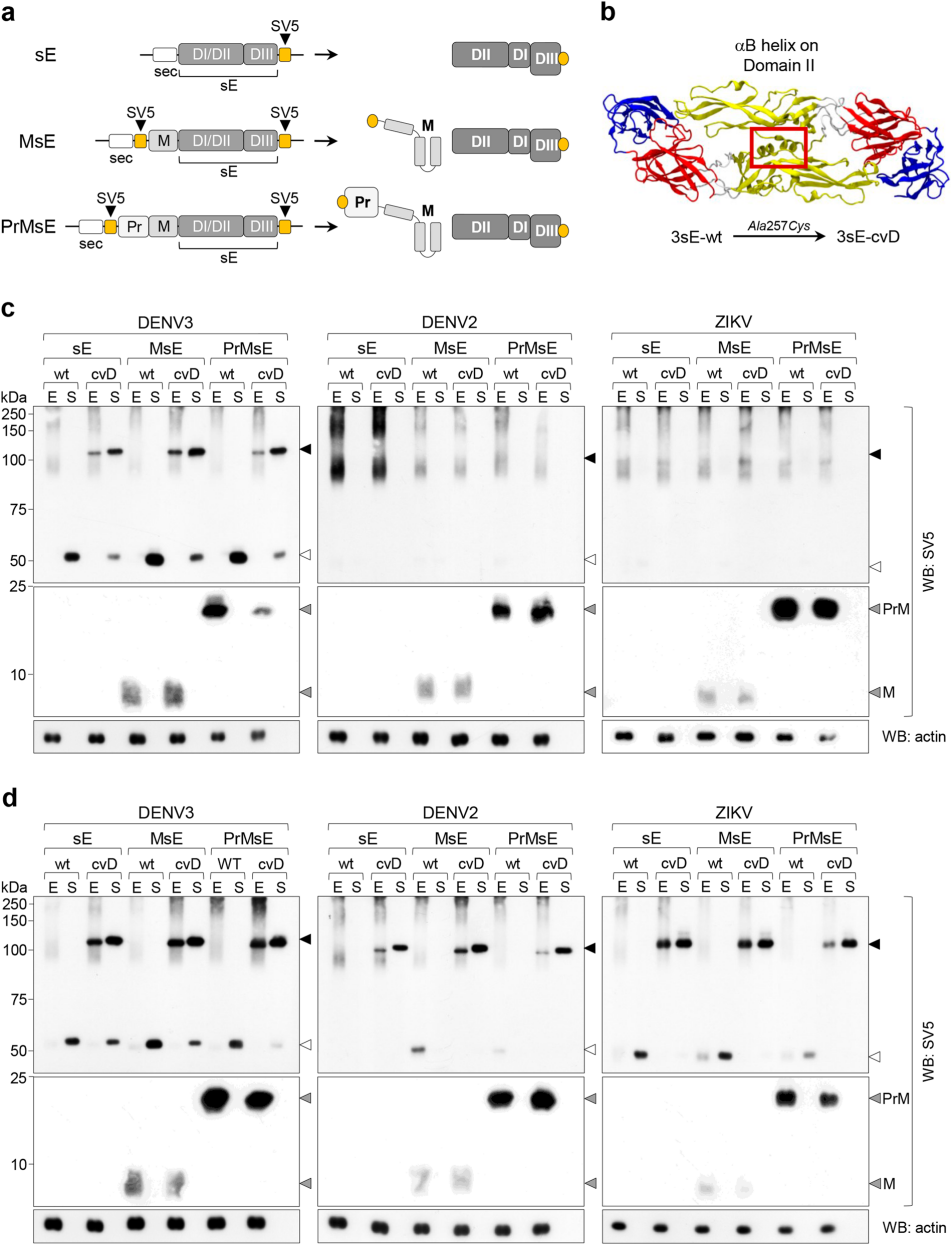
Dimerization and secretion of DENV and ZIKV sE proteins in mammalian cells. (**a**) Schematic representation of constructs and expected products. (**b**) Dimeric structure of DENV serotype 3 sE (PDB reference 1uzg) indicating the position of the αB helix where the Ala257Cys mutation was introduced. (**c**,**d**) Non-reducing western blots of total cell extracts (E) and culture supernatants (S) of HEK-293T cells transfected with the indicated constructs and incubated at 37 °C (**c**) or at 28 °C (**d**). Open and filled arrowheads indicate monomeric and dimeric sE, respectively.

Given that some of the most potent cross-neutralizing antibodies against DENV and ZIKV recognize complex quaternary epitopes that only exist within the context of the E dimer^28^, we attempted to stabilize the dimeric conformation of recombinant E from DENV and ZIKV expressed in mammalian cells. We have previously reported a detailed analysis of the secretory profile of recombinant E derived from the different DENV serotypes in mammalian cells and shown that DENV2 E ectodomain (2sE) undergoes different folding depending on temperature^36^. Here we have further investigated the role of temperature and PrM in the production and secretion of sE from DENV and ZIKV in transfected HEK293T cells, using constructs encoding sE alone or sE and the viral proteins PrM or only M (Fig. 1a). In addition, we introduced an Ala to Cys mutation in DII (Ala259 in DENV serotypes 1, 2 and 4, Ala257 in serotype 3 and Ala264 in ZIKV) because in the dimeric structure that residue is positioned just opposite of itself in the anti-parallel monomer (Fig. 1b). We reasoned that, if E undergoes transient dimerization in the ER, covalent stabilization through a disulphide bridge should be observed.

Non-reducing western blot (WB) of intracellular and secreted material from transfected HEK-293T cells incubated at either 37 °C or 28 °C (see protocol in Supplementary Fig. S1) are shown in Fig. 1c and d, respectively. At 37 °C, 3sE was secreted both as a monomer from wild-type (wt) constructs and as a covalent dimer (cvD) from constructs with the A257C mutation (Fig. 1c). Secretion and dimerization were independent of the presence of M or PrM as sE-wt and sE-cvD were equally produced and secreted in the presence or absence of PrM. In contrast, wt and cvD of both, 2sE and ZsE were not secreted regardless of the presence of M or PrM. A smear of disulphide bonded oligomers were detected intracellularly that most likely represent folding intermediates. At 28 °C (Fig. 1d), however, ZsE followed a secretory profile similar to 3sE and was efficiently produced and secreted both as sE-wt and sE-cvD, while 2sE was only secreted as disulphide-bonded dimers (2sE-cvD). Noteworthy, in both cases, the results were independent of the presence of PrM. 3sE-cvD showed similar dimerization and secretion profiles at 28 °C and 37 °C.

PrM-independent but temperature-dependent dimerization and secretion of cvD formats were also observed at 28 °C for DENV4 (4sE) and DENV1 (1sE) even though secretion of 1sE-cvD was less efficient (Supplementary Fig. S1). In addition, these characteristics were also independent of the mammalian cell line used (Supplementary Fig. S1).

**Figure 2:**
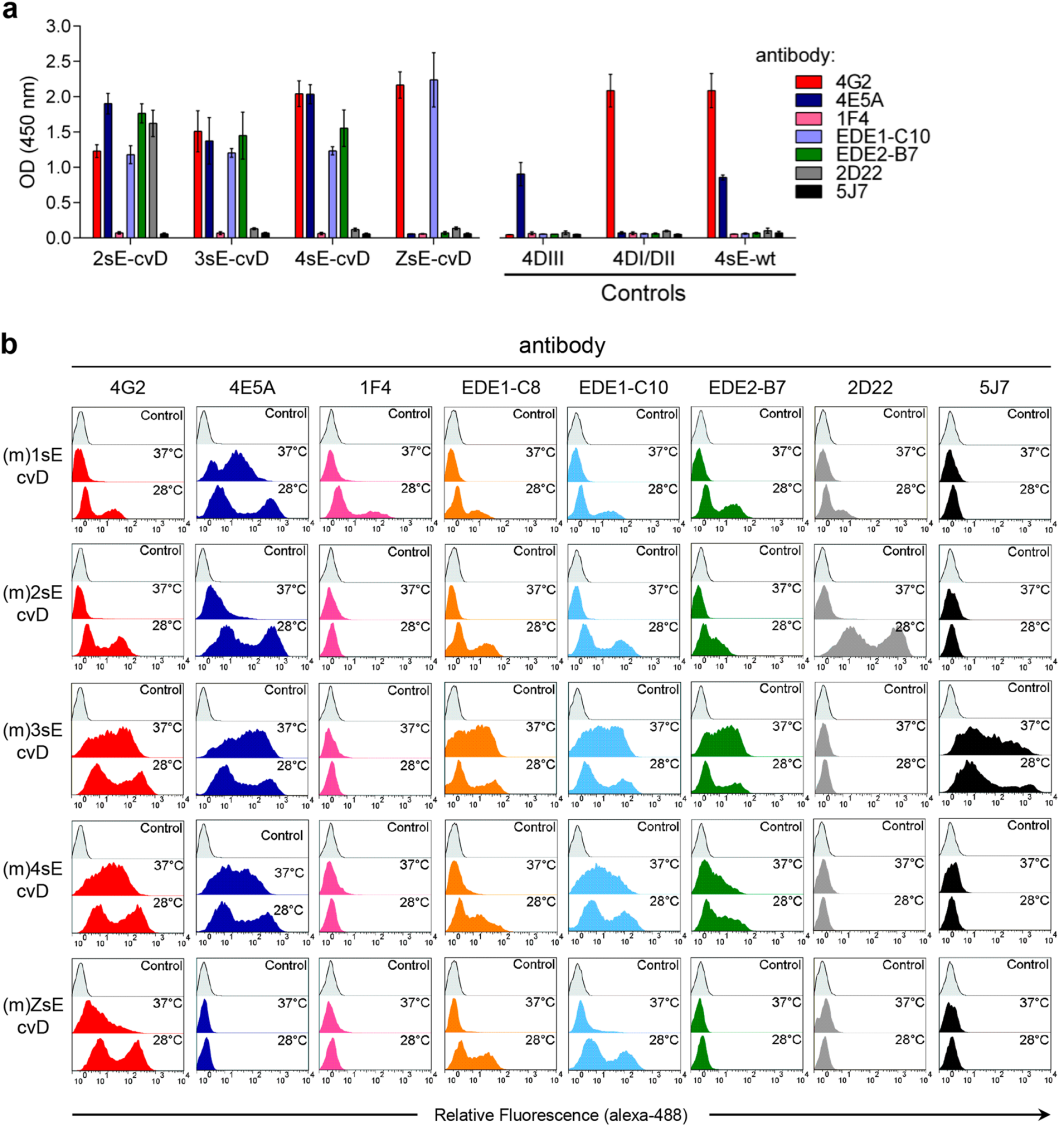
Structural analysis of covalently stabilized E dimers. (**a**) ELISA of sE-cvD from DENV serotypes 2, 3 and 4 and ZIKV with the indicated mAbs (left panel), data is represented as mean ± s.d. (for all mAbs, *n* = 5). Control antigens, 4DIII, 4DI/DII and 4sE wt were also reacted with the same set of antibodies (right panel), data is represented as mean ± s.d. (for all mAbs, *n* = 5). (**b**) Cytofluorimetric analysis of HEK-293T cells transfected with the membrane display sE-cvD versions of all four DENV serotypes and ZIKV at 37 °C or 28 °C and reacted with the indicated mAbs. Mock-transfected HEK-293T cells incubated with each mAb served as controls.

Correct folding and assembly of DENV and ZIKV sE-cvD was confirmed in ELISA and by cytofluorimetry of cells displaying dimers on the cell membrane. For ELISA, *in vivo* mono-biotinylated and secreted dimers of DENV 2, 3 and 4 (DENV1 sE-cvD is not secreted in sufficient amounts to perform a normalized assay) and of ZIKV were obtained from culture supernatants of transfected HEK-293T cells and immobilized on avidin-coated plates. We then tested different mAbs recognizing E conformational epitopes: 4G2 (fusion loop specific^37, 38^) and 4E5A (DENV DIII specific^39, 40^) recognize epitopes found on monomeric E, while EDE1-C10, EDE2-B7 (against E dimeric epitopes EDE1 and EDE2, respectively^27^), 2D22 (recognizing a DENV2-specific dimeric epitope^41^) and 5J7 (a DENV3-specific mAb that binds to 3 different E proteins on the viral surface^42^), bind to quaternary epitopes involving more than one E molecules. In addition, the DENV1- specific mAb 1F4 that binds to DI and the hinge region of a single E molecule, but only when present on the viral surface^43^, was also included. As shown in Fig. 2a, the EDE-specific mAbs EDE1-C10 and EDE2-B7 clearly detected cvD of 2sE, 3sE and 4sE while ZsE-cvD was detected by EDE1-C10 but not by EDE2-B7. As expected, 4G2 was positive in all cases, while 2D22 was positive only on 2sE-cvD and 4E5A recognized DIII of DENV but not of ZIKV. Not surprisingly, mAbs 1F4 and 5J7 were negative for all constructs, since the former binds only to DENV1, while the latter recognizes an epitope that requires a higher order arrangement of dimers.

The same set of reagents was used for cytofluorimetric analysis of cell membrane display of sE-cvD ((m)sE-cvD), achieved by fusing the trans-membrane and cytosolic tail of the type-I trans-membrane protein MHC-Iα at the C-terminus of sE, as previously reported^36^. Transfected cells were incubated with the set of mAbs following expression of (m)sE-cvD (Fig. 2b) or (m)sE-wt (Supplementary Fig. S2) at both, 28 °C and 37 °C. The profiles of reactivity resemble those of ELISA with three significant differences: i) the dimer-specific antibodies were able to detect the membrane displayed (m)sE-wt of the four DENV serotypes and ZIKV, indicating that they were present in the dimeric form, ii) mAb 1F4 recognized DENV1 E on both wt and cvD formats and iii) mAb 5J7 reacted similarly with DENV3 sE-cvD and sE-wt. An additional EDE1 specific mAb (EDE1-C8) was also included showing a profile similar to EDE1-C10. In agreement with recent reports, we found EDE1-type epitopes were conserved in ZIKV, while EDE2-B7 was not able to recognize ZIKV sE-cvD. Antibodies against EDE2-type epitopes have been shown to have a significantly reduced affinity towards ZIKV as binding is altered due to a change in the orientation of the glycan on DI (N154) and the lack of N-glycosylation in DII^28, 30^. This was demonstrated after analyzing a membrane displayed non-glycosylated ZIKV sE-wt mutant (Δglyc, T156I)^44^, which showed positive reactivity with mAb EDE2-B7 (Supplementary Fig. S3). As expected, reactivity to EDE1 mAbs was not affected.

We therefore concluded that both secreted and membrane bound sE-cvD, correspond to the dimeric structure present in mature viral particles.

### Viral particles with covalently stabilized E-dimers

**Figure 3:**
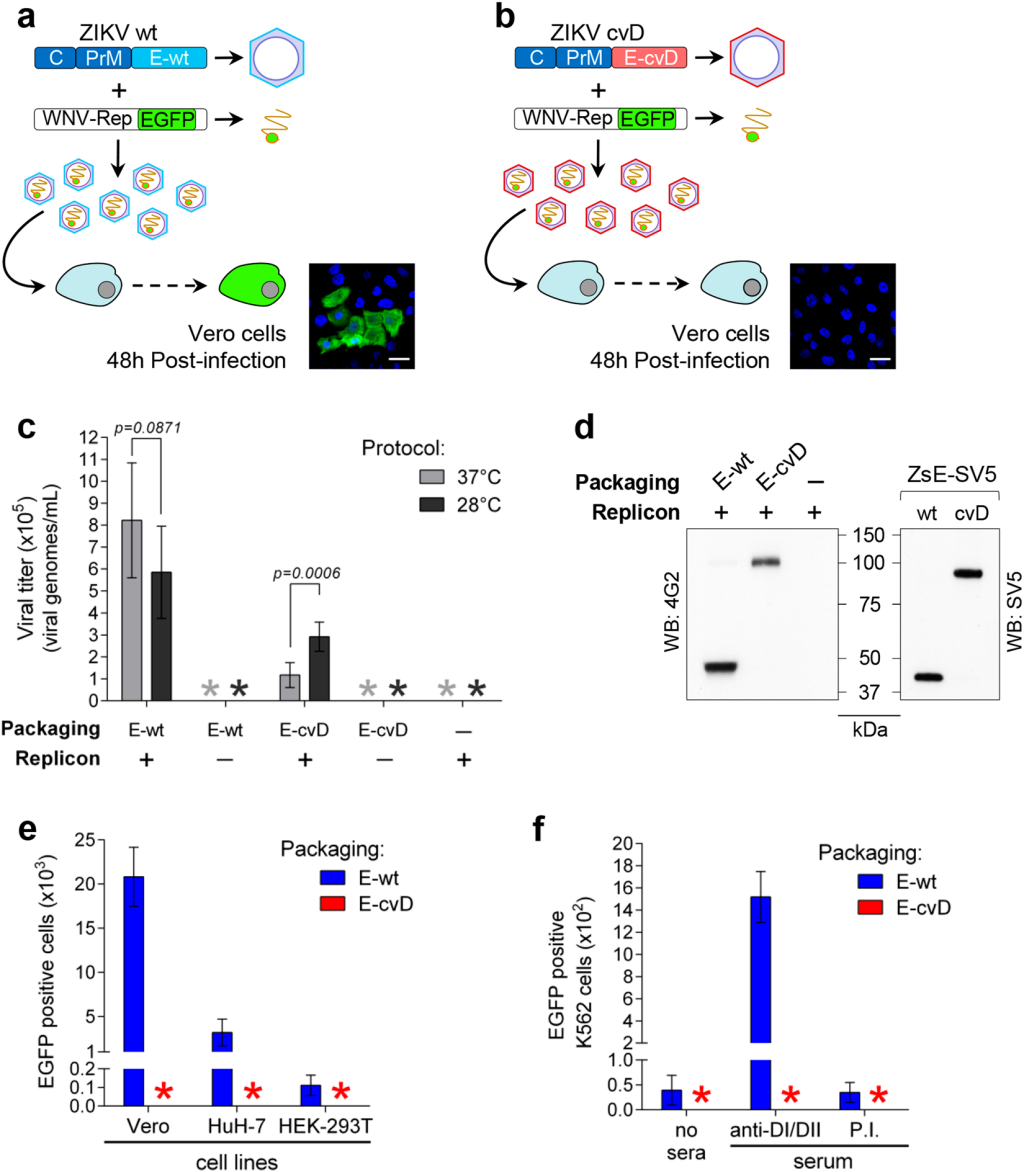
Pseudoviral particles packaged with ZIKV E-cvD protein. (**a**,**b**) Schematics of the ZIKV-wt (**a**) and -cvD (**b**) constructs and the WNV replicon used to produce pseudoviral particles, tested for infectivity (EGFP expression) on Vero cells. Images correspond to representative experiments (Bar, 30 μm). (**c**) Yield of pseudovirus determined by RT/PCR of the replicon-derived RNA, on samples obtained with ZIKV E-wt packaging construct with (for each temperature, *n* = 7; t = 1,863 df = 12) and without (for each temperature, *n* = 6) replicon, or ZIKV E-cvD packaging construct with (for each temperature, *n* = 6; t = 4,896 df = 10) and without replicon (for each temperature, *n* = 6), supernatant of cells transfected only with the WNV replicon was used as control (for each temperature, *n* = 6), data is represented as mean ± s.d. (**d**) Left panel, non-reducing Western blot of E protein from culture supernatants of cells transfected with WNV replicon and ZIKV E-wt or E-cvD packaging constructs, developed with mAb 4G2. Right panel, western blot of the corresponding sE SV5-tagged proteins developed with anti-SV5; migration differences correspond to the effect of the SV5 tag. (**e**) Infectivity of pseudoviral particles produced with ZIKV E-wt or E-cvD packaging constructs on Vero (for both preparations, *n* = 5), HuH-7 (for both preparations, *n* = 5) and HEK-293T cells (for both preparations, *n* = 5), determined as EGFP positive cells at 48 h post-infection (All cells were infected using 2 × 10^4^ viral particles as quantified by RT/PCR), data is represented as mean ± s.d. *, undetected. (**f**) Infectivity of same pseudoviral particles as in (**e**) on K562 cells in the absence of serum (for both preparations, *n* = 5) or in the presence of an anti-DI/DII serum (for both preparations, *n* = 5) or a negative control pre-immune serum (for both preparations, *n* = 5), data is represented as mean ± s.d. *, undetected.

We next attempted incorporation of the full-length covalent dimeric E into complete viral particles. For that purpose we generated pseudoviral particles by packaging a West Nile virus replicon encoding a reporter EGFP (WNV-rep)^45^ with a construct containing ZIKV-derived structural proteins C-PrME carrying the wild type sequence (E-wt) or the A264C cvD mutation (E-cvD). If assembled, particles with the cvD mutation should not be infective, as covalent E dimers would not allow E trimerization required for fusion of the viral envelope with endosomal membranes (Fig. 3a,b). Viral particles were obtained with both, E-wt and E-cvD mutant, as shown by RT/PCR amplification of replicon RNA from culture supernatants of cells co-transfected with E-wt or E-cvD and the WNV-rep, but not from cells transfected with the WNV-rep alone (Fig. 3c). While the production of pseudoviruses with E-wt was higher at 37 °C in some experiments (yet with no significantly different when comparing production at both temperatures), the yield of particles carrying the cvD mutation was significantly higher at 28 °C. Furthermore, the expected monomeric and dimeric forms of, respectively, E-wt and E-cvD were confirmed by non-reducing WB analysis of particles released in the culture supernatants at 28 °C (Fig. 3d). As expected, all the protein recovered in the E-cvD pseudoviral particles preparation was dimeric.

In contrast to pseudoviral particles with E-wt, those with E-cvD were not infective when tested in Vero, HuH-7 or HEK-293T cells (Fig. 3e). As previously mentioned, this could be due to impaired fusion with endosomal membranes or, alternatively, to impaired interaction with cellular receptors. In this latter case, E-cvD particles would be able to infect K562 cells when opsonized with anti-DI/DII antibodies, as Ab-mediated entry would be independent of viral receptors. As shown in Fig. 3f, while infectivity of particles packaged with E-wt was enhanced by anti-DI/DII antibodies, infectivity of those packaged with E-cvD was not, consistent with lack of E protein trimerization within endosomes due to the presence of the disulphide bond.

To our knowledge this is the first mutant pseudovirus with a dimeric covalent E protein described. The results indicate that virus particles with the E protein “frozen” in the dimeric conformation are permissive for assembly in the ER and compatible with trafficking through the secretory pathway.

### Hetero-dimerization of DENV and ZIKV sE

**Figure 4:**
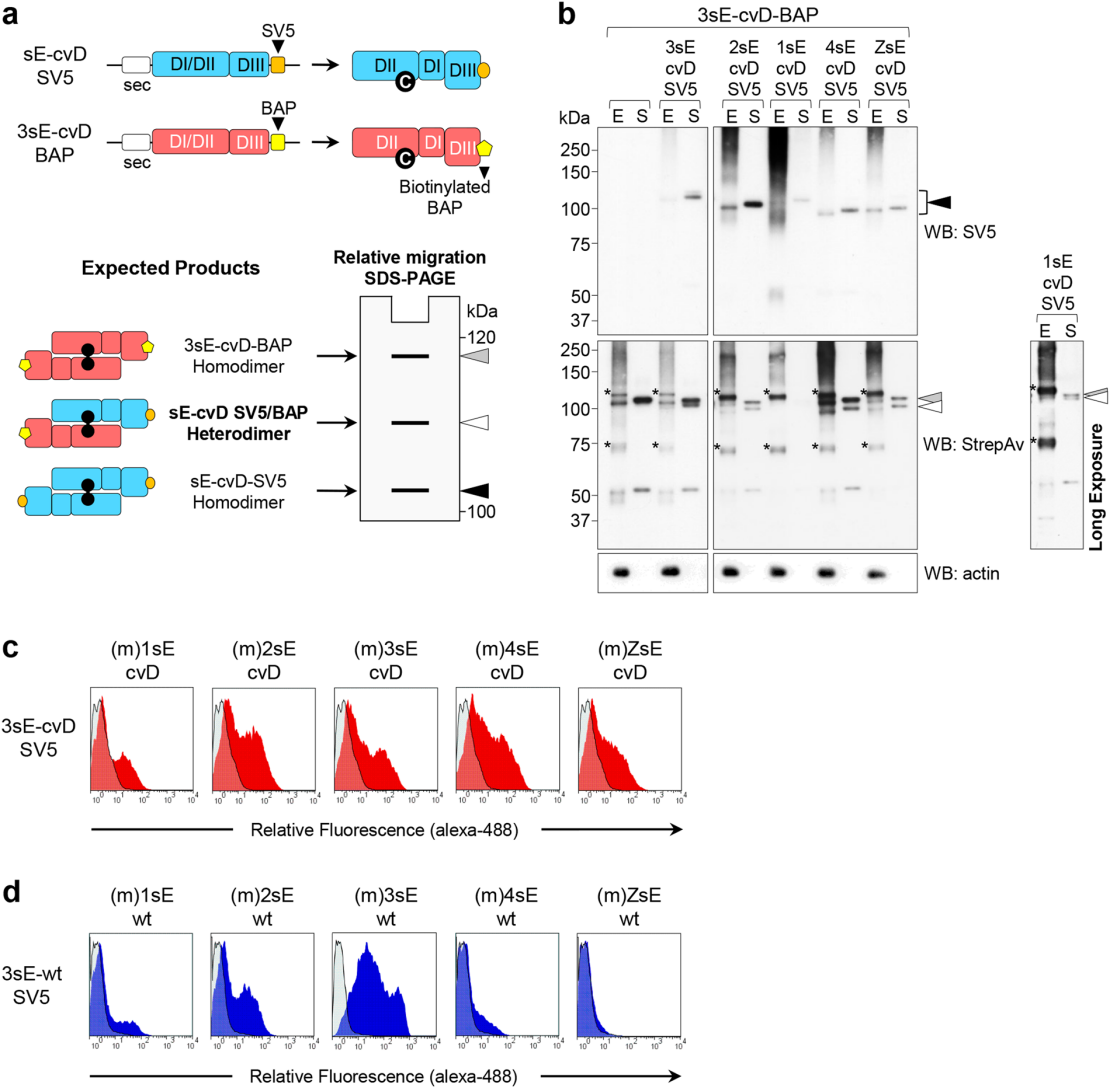
Hetero-dimerization of E proteins from different flaviviruses. (**a**) Schemes of sE-cvD constructs used. For all DENV serotypes and ZIKV, sE-cvD was SV5-tagged, while 3sE-cvD was also tagged with BAP and co-expressed with the SV5-tagged proteins. Expected homo- and hetero-dimers and their relative migration in non-reducing PAGE are indicated. (**b**) Non-reducing western blot of cell extracts (E) and culture supernatants (S) of HEK-293T cells co-transfected with the indicated constructs and developed with anti-SV5 (upper panel) or StrepAv-HRP (lower panel). *Indicates biotinylated host intracellular proteins. Arrowheads indicate migration of dimers as shown in (A). Open arrowheads indicate hetero-dimers. Right panel, long exposure of the poorly expressed 1sE-cvD-SV5 co-transfected with 3sE-cvD-BAP. (**c**,**d**) Cytofluorimetry of HEK-293T cells co-transfected with the membrane displayed (m)sE-cvD proteins (**c**) or (m)sE-wt proteins (**d**) of all four DENV serotypes or ZIKV and the secretory SV5-tagged 3sE-cvD (**c**) or 3sE-wt (**d**). Profiles correspond to anti-SV5 reactivity and are compared to HEK-293T cells transfected only with the secretory SV5-tagged 3sE.

Since all DENV and ZIKV sE harboring the cvD mutation were capable of forming and secreting dimers, we then asked whether interactions between sE belonging to different viruses were viable. To address this, we produced sE-cvD constructs with two different tags to allow discrimination of each monomer: 3sE was tagged with BAP (Biotin Acceptor Peptide, which can be biotinylated *in vivo* ^46, 47^) while 1sE, 2sE, 3sE (as a control), 4sE and ZsE were tagged with SV5^48^. Homo- and hetero-dimers were detected based on the different tags and the different gel migration (schematically shown in Fig. 4a). As shown in Fig. 4b, 3sE formed covalent hetero-dimers with 2sE, 4sE and ZsE that were secreted from HEK-293T cells. 1sE also formed hetero-dimers, although less efficiently (Fig. 4b, right panel).

These results were further confirmed by cytofluorimetry, after co-expression of the membrane-displayed (m)sE-cvD of each DENV serotype and ZIKV with the SV5-tagged secretory version of 3sE-cvD. Cells become positive for SV5 only when the secretory protein forms a hetero-dimer with the membrane-bound version (Fig. 4c). As expected, anti-SV5 staining was not observed when the secretory construct was expressed alone. Interestingly, although less efficiently in particular for DENV serotypes 1 and 4, hetero-dimers were also detected with the sE-wt proteins, while for ZIKV it was almost undetectable (Fig. 4d). This result confirms that hetero-dimerization takes place as a consequence of spontaneous interactions between different sE proteins, and is not imposed by the cvD mutation.

### E mosaic viruses

**Figure 5:**
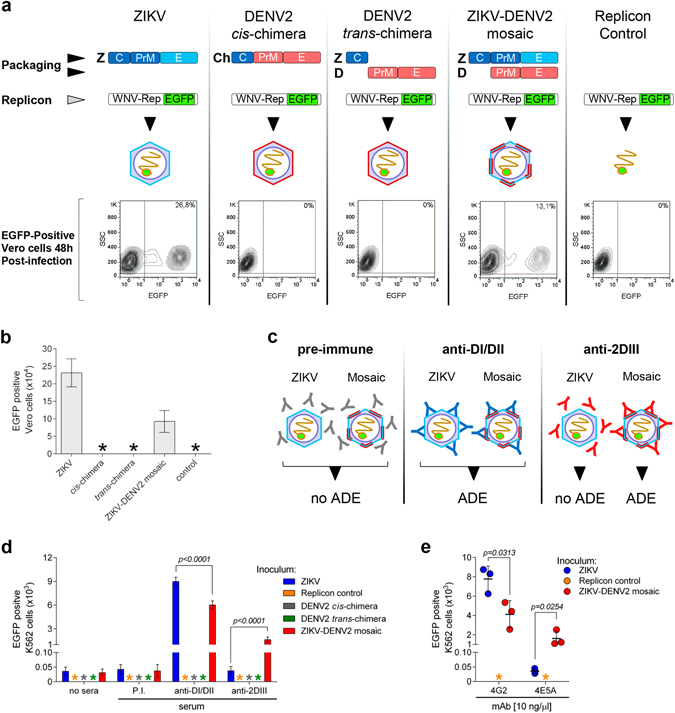
Assembly of ZIKV/DENV2 E protein mosaic pseudoviruses. (**a**) Pseudoviruses produced by co-transfection of the WNV replicon and different packaging constructs, were revealed by determining EGFP positive Vero cells following infection for 48 h. Percentages of EGFP positive cells on a representative sample are indicated. (**b**) Total number of infected Vero cells obtained using equal volumes of pseudoviral preparations as shown in (**a**) (for all preparations, *n* = 5), data is represented as mean ± s.d. (**c**) Scheme of the expected ADE activity for particles packaged with only ZIKV protein or ZIKV/DENV2 mosaic particles incubated with antibodies recognizing both, ZIKV and DENV2 E proteins (anti-DI/DII) or only DENV2 E protein (anti-2DIII). (**d**) Plot of EGFP positive K562 cells determined by cytofluorimetry, following infection of 5 × 10^4^ cells (for 48 h) with supernatants of transfections as in (**a**), and treated with anti-DI/DII (for all preparations, *n* = 5; t = 8,868 df = 8), anti-2DIII (for all preparations, *n* = 5; t = 10,70 df = 8) or control antibodies (*n* = 5). (**e**) Plot of EGFP positive K562 cells determined by cytofluorimetry, following infection of 5 × 10^4^ cells (for 48 h) with supernatants of transfections as in (**a**), and treated with mAb 4G2 (10 ng/μl) (*n* = 3; t = 3,252 df = 4) or mAb 4E5A (10 ng/μl) (*n* = 3; t = 3,478 df = 4). In all cases equal volumes of inoculum were used to infect cells, data is represented as mean ± s.d. *, undetected.

The hetero-dimerization results raised the possibility for distinct E proteins to assemble into viral particles with E mosaic structures. We thus investigated this further, asking whether it was possible to obtain pseudoviruses with the WNV-rep packaged with two different E proteins derived from ZIKV and DENV. Infective pseudoviral particles (revealed by Vero cells infection) were obtained with a full ZIKV construct (C^Z^-PrME^Z^) but not with a chimeric one containing ZIKV C protein and DENV2 PrME (C^Z^-PrME^D2^) (Fig. 5a, panels 1, 2). Transfection of the ZIKV C protein in *trans* with respect to DENV2 PrME did not produce infective particles, nor did transfection of the replicon construct alone (Fig. 5a, panels 3 and 5). In contrast, upon co-transfection of ZIKV C-PrME with DENV2 PrME, infective particles were produced (Fig. 5a, panel 4) although with a reduced efficiency compared to those obtained with only ZIKV proteins (Fig. 5b).

To test whether mosaic viral particles were produced, we used an ADE assay in which infection was mediated by antibodies specifically recognizing DENV2 E protein. By using antibodies highly specific for DENV2 (such as those directed against DIII^20^) infection should be enhanced for mosaic particles but not for those with only ZIKV E protein (Fig. 5c). As a control, antibodies against domains DI/DII cross-reacting with both DENV and ZIKV E proteins should produce ADE on both types of particles^20^ (Fig. 5c). As shown in Fig. 5d, particles produced from cells co-expressing the two E proteins were infective in K562 cells when opsonized with the DENV specific anti-2DIII serum, while the ones with only ZIKV E protein were not. Conversely, the cross-reacting anti-DI/DII antibodies produced ADE on both type of particles, while, as expected, a negative control serum did not (Fig. 5d). Further confirmation was obtained with mAb 4E5A, which is specific for DIII of DENV. As shown in Fig. 5e, 4E5A promoted infection of K562 cells of mosaic particles containing DENV E but not of control particles produced with only ZIKV protein. As expected, mAb 4G2 that recognizes the highly conserved FL, induced ADE on both types of particles. Thus, while DENV2 PrM-E protein alone was not able to package WNV-rep, it did get in part incorporated when co-expressed with the ZIKV proteins C-PrME, producing particles with a mosaic E composition.

## Discussion

Given the importance of complex quaternary epitopes for inducing potent neutralizing antibodies and based on our previous work regarding the secretory profile of DENV sE from mammalian cells^36^, we set out to analyze dimerization of DENV and ZIKV E protein. Although each of the proteins tested showed a characteristic secretory behavior, we found in all cases that intracellular dimerization of newly synthesized sE was highly dependent on temperature but independent of the presence of PrM.

At 28 °C, dimerization was highly favored for all DENV serotypes and ZIKV, resulting in complexes that could traffic and be secreted from mammalian cells. We could address this biochemical property of E because of the design of the Ala-Cys mutation in the alpha helix B (αB) of DII on the inner surface of the E monomer. In the dimeric configuration, the side chain of the inserted cysteine expands outwards, directly facing itself on the antiparallel monomer, which resulted in dimers covalently stabilized by a disulphide bond. This mutation was permissive for dimerization and secretion of all tested proteins. In addition, the mAbs included in the analysis allowed us to probe conformation of the structural domains as well as higher order interactions that mimic the quaternary structures found on the mature viral particles.

We showed that incubation at low temperatures was essential not only for secretion of the stabilized E dimers, but also for proper E folding. This observation is in line with recent reports showing that dengue virions suffer structural reconfiguration at temperatures above 33 °C^49, 50^. Our data represent, to our knowledge, the first report extending this temperature-dependent behavior to the secretory E protein from DENV serotypes and ZIKV. This information could be relevant when designing subunit vaccines.

We have previously reported that DENV serotypes showing sE poor secretory phenotypes were misfolded at 37 °C but properly folded at 28 °C^36^. Our data indicate that, while proper folding is heavily influenced by temperature, secretion of recombinant E proteins depends on their capacity to form relatively stable dimers as they transit through the ER and the secretory pathway. In fact, while for DENV3 and DENV4 sE secretion was observed for both wt and cvD forms regardless of the temperature, for DENV1 and ZIKV induction of proper folding at 28 °C was required and sufficient to achieve secretion of the wt protein. In contrast, secretion of DENV2 sE was only possible when the dimer was stabilized by the disulphide bond (despite being properly folded at 28 °C^36^) suggesting that wt dimers are relatively unstable and only allowed to traffic through the secretory compartment when covalently stabilized. This is consistent with recent structural data indicating that the surface of DENV2, and E dimeric interactions by consequence, is significantly more dynamic when compared to the other DENV serotypes^41^, which might explain the low stability of the 2sE-wt dimers.

Previous reports have shown enhanced production and stability of DENV and ZIKV virions at relatively low temperatures^51, 52^. However, it is difficult to extrapolate our observations on DENV and ZIKV E proteins and speculate on the effect of temperature on the dynamics of the viral cycle. Interactions with the ER membrane, as well as between viral and host proteins are likely to play pivotal roles on the propagation of the virus within the arthropod vector (28 °C) or the human host (37 °C) in the context of infection, and further studies are needed to understand the role of temperature in these scenarios.

The membrane display experiments further confirmed the role of temperature in proper folding of sE. sE-wt anchoring to the cell membrane favored stabilization of E dimeric conformations, most likely by keeping monomers in close proximity and thus resembling the dynamics that take place on the viral surface. Under these conditions (m)sE-wt constructs showed the same temperature-dependent behavior and structure as (m)sE-cvD constructs. These results indicate that dimerization of sE-wt is structurally equivalent to that of sE-cvD, and that the Ala-Cys mutation stabilizes the spontaneous interactions between E monomers.

With our set of reagents (both in ELISA and cell membrane display assays) we were able to discriminate the reactivity of antibodies based on different levels of structural complexity, from single domain specificity, like 4E5A on DIII or 4G2 on DII, to complex quaternary serotype-specific requirements, like 2D22. Moreover, reactivity of DENV3-specific mAb 5J7, which requires three different E molecules of two adjacent E dimers on the raft arrangement on the viral surface^41^, indicates that both (m)sE-cvD and (m)sE-wt are able to mimic complex structural arrangements beyond the dimeric E conformation. It is possible that assembly of quaternary epitopes is induced by the antibody upon binding, thus stabilizing the dimeric conformation (or higher structural arrangements) of (m)sE-wt proteins. This kind of antibody-mediated stabilization has been previously described for DENV sE proteins^27^.

Surprisingly, while temperature was found to be essential for sE dimerization and secretion, this was not the case for co-expression of PrM proteins. Many reports have established that PrM are important to assist folding of protein E in a chaperone-like manner^16, 53^. Even though there is compelling evidence highlighting the importance of PrM in preventing premature inactivation of E in the context of the whole viral particle^54^, the data regarding its chaperone-like function for the production and secretion of soluble E proteins are less convincing. In fact, previous studies have shown secretion of properly folded E in absence of PrM^36, 55, 56^, and our results indicate that this is also the case for soluble E dimers. This is important when evaluating E-based subunit or genetic vaccines against DENV or ZIKV, since most of them include PrM on the basis that it is required to achieve proper E folding, despite the fact that it has been proven that anti-Pr antibodies are rarely neutralizing and mostly enhance infection through ADE^57^. As proper quaternary E structures were obtained in absence of PrM, our data question its chaperon-like function and provide further evidence supporting its exclusion from future vaccine formulations.

The ZIKV E-cvD protein was capable of packaging the WNV replicon to form and secrete pseudoviral particles in which all E protein was present in the covalent dimeric form. Interestingly, our results show that, contrary to wild type pseudoviruses but in agreement with the results with the recombinant sE, production of particles with covalently dimerized E was significantly higher at 28 °C, further supporting the role of temperature in E-cvD folding. In addition, this shows that the Ala-Cys mutation and the consequent disulphide bond are compatible with the higher order arrangements present on the viral surface. The data indicate that E dimerization occurs shortly after translation and that transport and secretion of full particles with covalently stabilized dimers are allowed; it also suggests that the “spiky” arrangement of E on newly assembled immature particles within the ER lumen is not strictly required^58, 59^.

As expected, E-cvD particles were not infective, highlighting the need of E trimerization within endosomes at the low pH conditions that promote fusion of the viral envelope with endosomal membranes^60^. In addition, our data support the idea that viral neutralization can be induced by interfering with viral surface dynamics, a mechanism that has been suggested to explain the strong neutralization showed by antibodies targeting the hinge region or the complex quaternary epitopes^41, 42^, recently demonstrated for ZIKV neutralisation by mAb EDE1-C10^61^.

The ability of sE proteins from different DENV serotypes and ZIKV to form hetero-dimers was surprising. We confirmed this initial observation in the context of pseudoviruses assembled into phenotypically mixed mosaic particles carrying ZIKV and DENV2 E proteins. Although we lack the means to determine the precise structural configuration and composition of these particles, their mosaic nature was shown by promoting ADE on K562 cells with an anti-DENV2 specific polyclonal serum and with the DENV-specific mAb 4E5A.

Phenotypically mixed viruses have been previously produced for Influenza A/B viruses^62^. Here we show for the first time that mosaic particles with E proteins from two different flaviviruses can assemble into infective particles. Although single cell flaviviral co-infection has not been reported, our data suggest that in such scenario particles with enhanced infectivity in the presence of antibodies specific for either of the two different E proteins could exist.

Even though antibodies are important to neutralize the virus, the specificity required for efficient protection is still debated. Latest studies indicate that antibodies against DIII and complex quaternary epitopes of E, but not those against DI or DII, are able to strongly neutralize the infection with low risk of ADE, making these antigens preferable for vaccine developments^20, 30, 34^. The ELISA and cytofluorimetry-based assays presented here provide efficient tools not only to study the binding properties of antibodies, but also to screen for those able to recognize dimeric and more complex epitopes without the safety concerns of using whole viral preparations. Moreover, our covalent stabilized E dimers represent a first step to exploit the immunogenic potential of quaternary epitopes and formulate efficient cross-neutralizing vaccines.

## Materials and Methods

### Cell lines

HEK-293T/17 (HEK-293T, ATCC, Rockville, MD, USA, number CRL-11268), Vero (ATCC CCL-81), HuH-7 (kindly provided by Dr. Alessandro Marcello, ICGEB, Trieste, Italy) and HeLa cells (ATCC CCL-2) were cultured in Dulbecco’s modified Eagle’s medium (DMEM, Life Technologies, Paisley, UK) supplemented with 10% heat-inactivated foetal calf serum (FCS) (Life Technologies), 50 μg/ml gentamycin and 2 mM L-glutamine. K562 cells (ATCC CCL-243) were maintained in RPMI 1640 medium (Life Technologies) supplemented with 10% FCS and 50 μg/ml gentamycin. Cell cultures were grown at 37 °C with 5% of CO_2_. Mycoplasma contamination was excluded by PCR.

### Plasmid DNA constructs

Synthetic fragments (obtained from GenScript, Piscataway, NJ, USA) containing the codon optimized PrM-sE sequences from DENV2 New Guinea C strain (GenBank accession number AF038403, aminoacids 117-675 of the viral polyprotein), DENV3 3H87 strain (GenBank accession number M93130, aminoacids 118-673) and ZIKV Mr766 strain (GenBank accession number AEN75266.1, aminoacids 122-694), and sE sequences form DENV1 Nauru Island strain (GenBank accession number U88535.1, aminoacids 281-675) and DENV4 Dominica strain (GenBank accession numbers AF326573.1, aminoacids 280-674) were obtained and fused to an amino-terminal immunoglobulin leader sequence (sec)^63^ and to a carboxy-terminal SV5 tag (GKPIPNPLLGLD)^48^ in pVAX vectors (Life Technologies). MsE-SV5 and sE-SV5 constructs from DENV2 (aminoacids 206-675, and 281-675, respectively), DENV3 (aminoacids 206-673, and 281-673, respectively) and ZIKV (aminoacids 216-694, and 291-694, respectively), were obtained by removing the Pr or PrM coding regions, respectively. Non-glycosylated ZIKV sE mutant (GenBank accession number AHL43505.1) were obtained by site-directed T156I mutagenesis (QuikChange XL Site-Directed Mutagenesis Kit, Agilent Technologies, La Jolla, CA, USA) as previously described^44^.

Constructs tagged with BAP (biotin acceptor peptide, GLNDIFEAQKIEWHE)^46, 47^, or targeted for membrane display (with the transmembrane and cytoplasmic domains of the human MHC-Iα chain), were obtained by cloning into the corresponding vectors as previously reported^36^.

For pseudovirus production, codon optimized sequences coding for C-PrM-E genes of ZIKV Mr766, and the chimera containing the C gene from ZIKV Mr766 and the PrM-E form DENV2 New Guinea C strain (C^Z^-PrME^D2^), were obtained as synthetic fragments and cloned into pVAX vectors. Plasmids expressing only C from ZIKV or PrME from DENV2 were obtained by deletion the PrME and C coding regions, respectively.

Covalent E dimers were obtained by site-directed mutagenesis (Ala to Cys) of Ala259 for DENV1, 2 and 4, Ala257 for DENV3 and Ala264 for ZIKV (QuikChange XL Site-Directed Mutagenesis Kit, Agilent Technologies, La Jolla, CA, USA) following manufacturer instructions.

### Monoclonal antibodies and mouse sera

Purified human mAbs 2D22^41^, 1F4^43^ and 5J7^42^ as well as mouse mAb 4G2^37^, were kindly provided by Prof. Aravinda de Silva (University of North Carolina, Chapel Hill, NC, USA). Mouse mAb 4E5A^40^ was kindly provided by Prof. Jonathan R. Lai (Albert Einstein College of Medicine, NY, USA). mAbs EDE1-C8 (clone 752-2 C8), EDE1-C10 (clone 753(3) C10) and EDE2-B7 (clone 747 B7) were constructed as human IgG antibodies from previously deposited sequences^27^ and used as culture supernatants from transfected cells. mAb anti-SV5^48^ was obtained as previously described^64^. Two groups of six 5-weeks old, female Balb/c mice (Harlan, Milan, Italy) were immunized three times at two weeks intervals by Gene Gun technology (Bio-Rad, Hercules, CA, USA) using 1 μm gold particles coated with 1 μg of plasmid DNA encoding either the domain DIII of DENV2 E protein or domains DI/DII of DENV4 E protein as previously described^56^. Blood samples were collected at day 90 by sub-mandibular puncture and pooled to obtained anti-2DIII and anti-4DI/DII sera, respectively. Pooled sera from non-vaccinated animals were used as negative control (Pre-immune sera). All animal procedures described in this study were approved by the ICGEB Animal Welfare Board and the Italian Ministry of Health (Ministero della Salute) (protocol DGSAF0024706) and conducted in accordance to international and institutional guidelines for animal experimentation, and in compliance to laws and policies established in the legislation D. L.vo 26/2014 of the Italian Government.

### Expression of secretory E proteins

Standard calcium phosphate transfections were performed on HEK-293T cells as previously described^65^. For Vero and HeLa cells, transfections were done with Lipofectamine 3000 (Life Technologies) following manufacturer’s protocols. After overnight incubation at 37 °C, culture medium was replaced with a serum-free medium and cells incubated for another 24 h at 37 °C or 28 °C; when required media was supplemented with 100 μM biotin (Sigma-Aldrich, St. Louis, MO, USA). Free biotin was removed from cultures supernatants by dialysis against PBS. For hetero-dimerization assays SV5-tagged and BAP-tagged constructs were co-transfected in a 2:1 ratio. In all cases, cellular extracts were prepared in 100 μl of TNN lysis buffer (100 mM Tris-HCl, pH 8, 250 mM NaCl, 0.5% NP-40) at 4 °C, supplemented with Protease Inhibitor Cocktail (Sigma-Aldrich).

### Production of Pseudoviral particles

Pseudoviral particles were produced by co-transfection of a DNA-launched WNV replicon expressing EGFP^45^ (kindly provided by Dr. Theodore Pierson, National Institute of Allergies and Infectious Diseases, MD, USA) with plasmids expressing the structural genes of ZIKV or DENV2 in *trans*, as previously described^34^. Briefly, HEK-293T cells were transfected with linear polyethylenimine (MW 25,000, PEI, Polysciences, Warrington, PA, USA). After 16 h, culture medium was replaced with a DMEM-7% FCS and incubation was extended for another 24 h at 37 °C or 28 °C. Pseudoviruses were harvested, clarified from cell debris by centrifugation (10 min at 10,000 rpm) and stored at −20 °C until use. For western blot analysis, particles were concentrated by ultracentrifugation at 40,000 g for 24 h.

### Quantification of pseudoviruses by Real-Time PCR

WNV Replicon-derived RNA was isolated from 100 μl of pseudoviral preparations using the RNAzol-BEE solution (Tel-Test, Friendswood, TX, USA) and then treated with DNaseI (Promega, Madison, WI, USA) following manufacturer’s instructions. For cDNA synthesis, reverse transcription was performed using random hexamers (Sigma) and M-MLV Reverse transcriptase (Life Technologies) according to the manufacturer’s protocol. The product of retrotranscription was used as a template for quantitative real-time PCR using previously reported 3′UTR specific primers^66^ and EvaGreen Technology (Bio-Rad, Hercules, CA, USA) on a C1000 thermal cycler using CFX96™ Real-Time Detection System (Bio-Rad). Quantification of the relative number of viral RNA genomic copies was calculated by interpolating in a standard curve made with a quantified WNV-rep plasmid (r^2^ = 0.9972). This method was used in pseudoviral infection assays to determine the multiplicity of infection (MOI).

### Western blot

Samples were separated by non-reducing 10% SDS-PAGE, transferred to polyvinylidene difluoride (PVDF) membranes (Millipore, Temecula, CA, USA) and blocked a 5% milk solution in PBS (PBS-milk). When detecting SV5-tagged proteins, membranes were incubated for 1 h with anti-SV5 mAb (1 μg/ml), washed, and probed with HRP-linked anti-mouse IgG goat antibodies (KPL, Gaithersburg, MA, USA, 074–1809, 1:10,000) for 1 h. In the case of biotinylated proteins, the membranes were probe with HRP-linked streptavidin (Jackson ImmunoResearch, Newmarket, UK, 016-030-084, 1:20,000) for 1 h after blocking. For detection of pseudo viral particles, membranes were incubated with mAb 4G2 (40 ng/μl in PBS-milk) for 1 h, washed, and treated with HRP-linked anti-mouse IgG goat antibodies as before. Mouse HRP-conjugated anti-actin mAb (clone AC-15, Sigma-Aldrich, 1:30,000) was used as loading control. Signals were developed by ECL (ThermoFisher-Pierce, Rockford, IL, USA).

### ELISA

Mono-biotinylated BAP-tagged proteins were normalized by western blot and captured overnight on Nunc Maxi Sorp Immuno-Plates (ThermoFisher-Nunc, Roskilde, Denmark) pre-coated with 100 μl/well of 5 μg/ml avidin (Sigma-Aldrich) in 50 mM Na_2_CO_3_/NaHCO_3_ buffer as previously described^56^. After washing, plates were incubated for 1 h with 100 μl/well of the different mAbs (mAb 4G2, 0.15 ng/μl; mAb 4E5A, 0.5 ng/μl; mAb 2D22, 0.5 ng/μl; mAb 1F4, 0.5 ng/μl; mAb 5J7, 0.5 ng/μl; mAbs EDE1-C8, EDE1-C10 and EDE2-B7 were used as undiluted supernatants). After washing, 100 μl/well of HRP-linked anti-mouse IgG γ-chain goat antibodies (Jackson ImmunoResearch, 115-035-071, 1:50,000) or HRP-linked anti-human IgG goat antibodies (KPL, 074-1002, 1:20,000) were added according to the mAb used, and incubated for 1 h at RT. The bound conjugate was detected using TMB substrate (Sigma), stopped with H_2_SO_4_ 1 M and measured at 450 nm (OD_450_) on a Bio-Rad iMark microplate reader.

### Cytofluorimetry

HEK293T cells were transfected with constructs encoding membrane-displayed versions of sE-wt or sE-cvD proteins, as described before^36^. Cells were then washed and resuspended in PBS containing 3% BSA (Sigma-Aldrich) and 5 μM EDTA, incubated with the different mAbs (mAb 4G2, 1 ng/μl; mAb 4E5A, 1 ng/μl; mAb 2D22, 1 ng/μl; mAb 1F4, 1 ng/μl; mAb 5J7, 1 ng/μl; mAbs EDE1-C8, EDE1-C10 and EDE2-B7 were used as undiluted supernatants) for 1 h at RT followed by Alexa488-conjugated goat anti-mouse IgG (Jackson ImmunoResearch, 115-545-062, 1:1,000) or Alexa488-conjugated goat anti-human IgG (Jackson ImmunoResearch, 109-545-003, 1:1,000), and analyzed in a FACSCalibur (BD Biosciences, San Jose, CA, USA). For hetero-dimerization assays, secretory SV5-tagged 3sE (−wt or –cvD) and membrane displayed proteins were co-transfected at a 2:1 ratio, and incubated with anti-SV5 (1 μg/ml) for 1 h.

### Immunofluorescence

Vero cells were infected with pseudoviral preparations at multiplicity of infection (MOI) of 0.1. 48 h post-infection cells were fixed with 3.7% paraformaldehyde (Sigma-Aldrich) in PBS for 20 min and quenched with 150 mM glycine in PBS. After washing, cells were mounted with ProLong supplemented with DAPI (Thermo Fisher Scientific). Images were acquired using a Nikon Eclipse Ti-E microscope.

### Infection of mammalian cells

24 h before infection cells were seeded at a density of 5 × 10^4^ cells/well in 24-well plates. For infection, pseudoviral preparations were diluted in serum-free DMEM and added to each well for 3 h at 37 °C, 0.5 ml of DMEM with 2% FBS was then added and plates further incubated for 48 h. After incubation, cells were resusupended, counted and analyzed for EGFP expression by cytofluorimetry.

### Antibody-dependent pseudoviral infection of K562 cells

Equal volumes of 1:100 serum dilutions, mAb 4G2 (10 ng/μl) or mAb 4E5A (10 ng/μl) and different preparations of pseudoviral particles were mixed and incubated for 1.5 h at 36 °C in round-bottom 96 multi-well plates (Corning-Costar, Corning, NY, USA). 5 × 10^4^ K562 cells were added to the serum-virus mixture and incubated for 48 h at 36 °C. Cells were then washed, resuspended in PBS and analyzed for EGFP expression by cytofluorimetry.

### Statistical analysis

Data was obtained from at least three independent experiments done in duplicate or triplicate. Unless indicated otherwise, arithmetic means ± standard deviations were calculated. Unpaired two-tailed *t* test was used for dual comparison analyses when needed (GraphPad Prism 6.0, GraphPad Software Inc., La Jolla, CA, USA); in all cases *p* values < 0.05 were considered significant and variances between compared groups were not significantly different (p > 0.05 using F test). Sample size was not statistically assessed and data distribution was assumed to be normal. No randomization was done. Investigators were not blind for data collection or analysis.

### Data availability

The authors declare that the data supporting the findings of this study are available within the article and its Supplementary Information or can be obtained from the corresponding author upon request.

## Electronic supplementary material

Supplementary Information
