# Supplementary material for: Temperature-dependent folding allows stable dimerization of secretory and virus-associated E proteins of Dengue and Zika viruses in mammalian cells
Source: Sci Rep. 2017 Apr 19;7:966. doi: 10.1038/s41598-017-01097-5 (PMC5430527; doi:10.1038/s41598-017-01097-5)
Supplement: Supplementary file 1 — Supplementary Information [file 41598_2017_1097_MOESM1_ESM.pdf]

Supplementary Information

Temperature-dependent folding allows stable dimerization of secretory and virus-associated E proteins of Dengue and Zika viruses in mammalian cells

J.L. Slon Campos[a,], S. Marchese[a] , J. Rana[a] , M. Mossenta[a], M. Poggianella[a,], M. Bestagno[a], O.R. Burrone[a,*]

[a] Molecular Immunology Group, International Centre for Genetic Engineering and Biotechnology, 34149 Trieste , Italy

[*] Address correspondence to Oscar R. Burrone, burrone@icgeb.org, tel: +39-040-3757314, Trieste, Italy.

Supplementary Figure S1

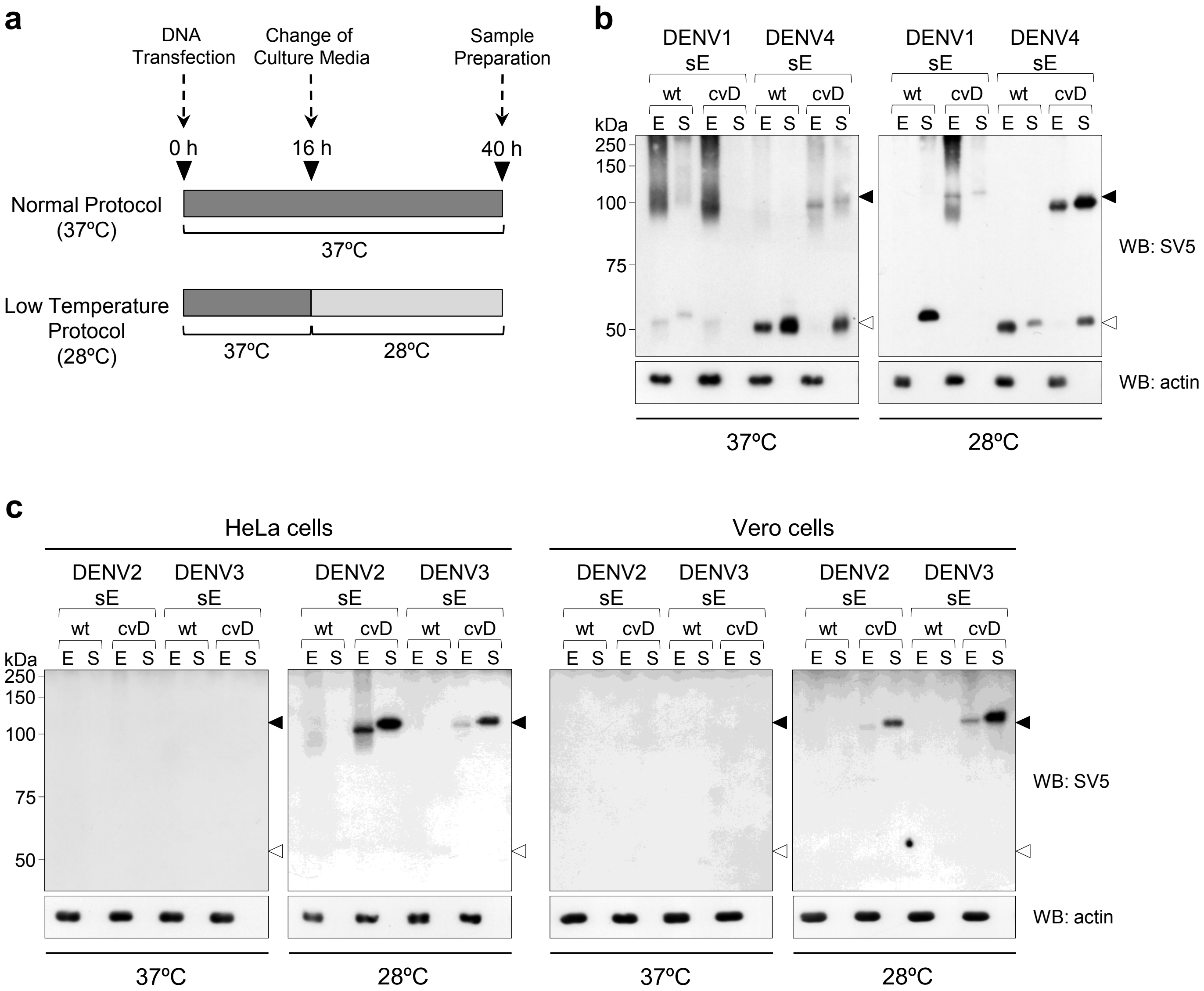

Supplementary Figure S1. Dimerisation and secretion of sE proteins in mammalian cells. a) Scheme of the experimental conditions after transfection and temperature shifts. **b)** Non-reducing western blots of total cell extracts (E) and culture supernatants (S) of HEK-293T cells transfected with sE-wt and sE-cvD constructs of DENV1 and DENV4, incubated at 37ºC (left panel) or at 28ºC (right panel). Open and filled arrowheads indicate monomeric and dimeric sE, respectively. **c)** Dimerisation and secretion of sE-wt and sE-cvD of DENV2 and DENV3 in HeLa (left) or Vero cells (right), incubated at 37ºC or 28ºC, as indicated.

Supplementary Figure S2

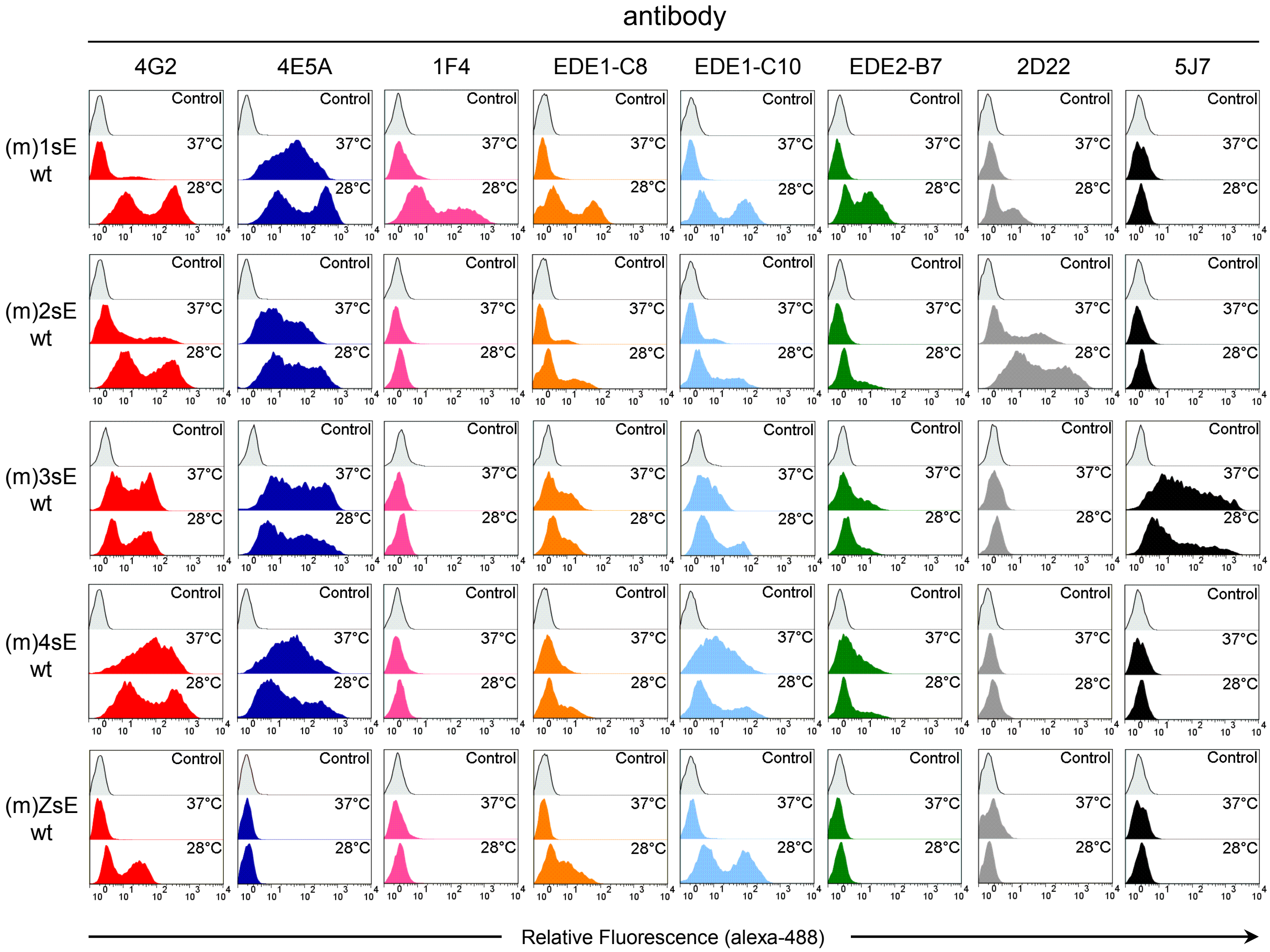

Supplementary Figure S2. Structural analysis of sE-wt proteins from DENV and ZIKV. Cytofluorimetric analysis of HEK-293T cells transfected with the membrane display sE-wt versions of all four DENV serotypes and ZIKV at 37ºC or 28ºC and reacted with the indicated mAbs. Mock-transfected HEK-293T cells incubated with each mAb served as controls.

Supplementary Figure S3

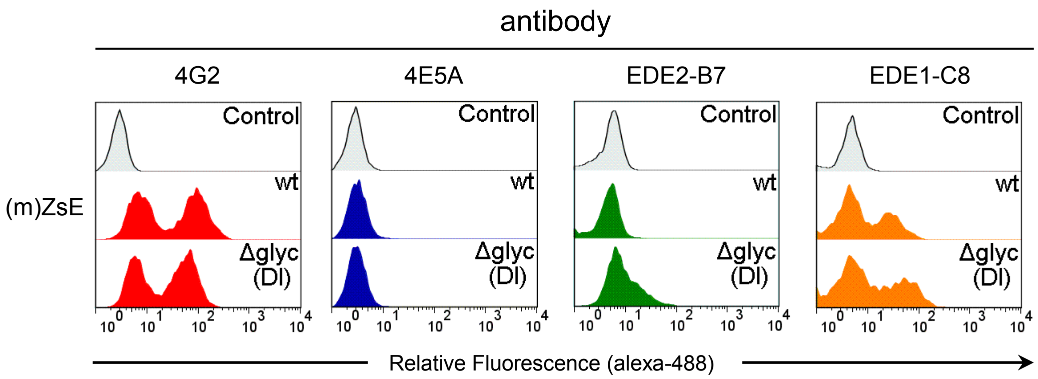

Supplementary Figure S3. Effect of E glycosylation on EDE2 B7 reactivity. Cytofluorimetric analysis of HEK-293T cells transfected at 28ºC with the sE membrane display construct of ZIKV wt or the non-glycosylated mutant (Δglyc) on domain I (T156I), and reacted with the indicated mAbs. Mock-transfected HEK-293T cells incubated with each mAb served as controls.
